# A genetic framework for H_2_O_2_ induced cell death in *Arabidopsis thaliana*

**DOI:** 10.1186/s12864-015-1964-8

**Published:** 2015-10-23

**Authors:** Eve Kaurilind, Enjun Xu, Mikael Brosché

**Affiliations:** Division of Plant Biology, Department of Biosciences, Viikki Plant Science Centre, University of Helsinki, Helsinki, Finland; Institute of Ecology and Earth Sciences, University of Tartu, Tartu, Estonia; Institute of Technology, University of Tartu, Nooruse 1, Tartu, 50411 Estonia

**Keywords:** Programmed cell death, Reactive oxygen species, Salicylic acid, Jasmonic acid, Auxin, Lesion mimic mutants

## Abstract

**Background:**

To survive in a changing environment plants constantly monitor their surroundings. In response to several stresses and during photorespiration plants use reactive oxygen species as signaling molecules. The *Arabidopsis thaliana catalase2* (*cat2*) mutant lacks a peroxisomal catalase and under photorespiratory conditions accumulates H_2_O_2_, which leads to activation of cell death.

**Methods:**

A *cat2* double mutant collection was generated through crossing and scored for cell death in different assays. Selected double mutants were further analyzed for photosynthetic performance and H_2_O_2_ accumulation.

**Results:**

We used a targeted mutant analysis with more than 50 *cat2* double mutants to investigate the role of stress hormones and other defense regulators in H_2_O_2_-mediated cell death. Several transcription factors (AS1, MYB30, MYC2, WRKY70), cell death regulators (RCD1, DND1) and hormone regulators (AXR1, ERA1, SID2, EDS1, SGT1b) were essential for execution of cell death in *cat2*. Genetic loci required for cell death in *cat2* was compared with regulators of cell death in spontaneous lesion mimic mutants and led to the identification of a core set of plant cell death regulators. Analysis of gene expression data from *cat2* and plants undergoing cell death revealed similar gene expression profiles, further supporting the existence of a common program for regulation of plant cell death.

**Conclusions:**

Our results provide a genetic framework for further study on the role of H_2_O_2_ in regulation of cell death. The hormones salicylic acid, jasmonic acid and auxin, as well as their interaction, are crucial determinants of cell death regulation.

**Electronic supplementary material:**

The online version of this article (doi:10.1186/s12864-015-1964-8) contains supplementary material, which is available to authorized users.

## Background

During their life cycle plants can experience multiple stress conditions – ranging from abiotic stresses (including fluctuating temperatures, water availability and light intensity) to biotic stresses (pathogen infection, insect attack). The early steps of plant defense against pathogens include recognition of conserved structures of the pathogen and activation of downstream responses [1]. In contrast, little information is available about the initial perception mechanisms during abiotic stresses. Down-stream from initial stress perception several signaling molecules have been identified, including changes in cytosolic Ca^2+^ concentration, production of reactive oxygen species (ROS) and activation of hormone signaling pathways including salicylic acid (SA), jasmonic acid (JA), ethylene and abscisic acid (ABA) [2]. Furthermore it is clear that defense signaling is not organized in linear parallel pathways, instead hormones have both synergistic and antagonistic interactions [2]. Consequently, the outcome of a stimulus is not determined by a single signaling molecule but a “web of interactions” involving multiple signaling pathways.

One of the early events after stress perception is the enhanced production of ROS, such as superoxide (O_2_^**.**-^) and hydrogen peroxide (H_2_O_2_) that are used as signaling molecules [3]. ROS signaling in turn alters the redox state, initiates Ca^2+^ signaling, activates protein kinases and cellular metabolic pathways [3]. Conversely, abiotic stress can alter the cell redox state which ultimately lead to increased ROS production [4]. Furthermore, ROS are produced in different cellular compartments which can activate separate signaling pathways [5]. This makes it important to distinguish the subcellular localization of ROS production and how these molecules integrate into other signaling pathways, including the regulation of programmed cell death (PCD) and changes in gene expression. Generally, ROS are removed by ROS scavenging enzymes including catalase and low molecular weight antioxidants including ascorbate and glutathione [4]. Catalases are localized in peroxisomes and detoxify H_2_O_2_ produced from glycolate oxidation [6]. Glycolate is formed during photorespiration when Rubisco uses O_2_ instead of CO_2_. It is estimated that a majority of H_2_O_2_ in the cell is produced during photorespiration and photosynthesis [7]. This makes photorespiration an important contributor to cellular redox state and this process may help protect cells and provide adaption to unfavorable conditions [8]. The importance of the interaction between ROS from different sources in defense signaling is reinforced by the study of plants deficient in both ASCORBATE PEROXIDASE and CATALASE, which are surprisingly more tolerant to different environmental conditions than plants deficient in only one scavenger [9]. Thus, ROS signaling is intimately connected to redox signaling as well as to the regulation of PCD [10].

PCD is an integral part of plant life and regulate stress, growth and developmental responses. Furthermore, in defense against pathogens, PCD at the site of infection known as the hypersensitive response (HR), is correlated with increased resistance to some pathogens [10]. Forward genetic screens in *Arabidopsis thaliana* have identified mutants with misregulated or spontaneous cell death. These mutants, called lesion mimic mutants (LMMs), have altered cell death phenotypes, which are usually accompanied by altered responses to pathogen infection [11, 12]. Since several LMMs exhibit large visible lesions on leaves and/or are dwarfs, they have been used in suppressor mutant screens or reverse genetics screens to identify additional regulators of PCD [11, 12]. SA has a central role in regulation of cell death. Many LMMs accumulate high amounts of SA and introduction of the bacterial enzyme salicylate hydroxylase (*NahG*) that degrades SA significantly reduces the extent of cell death in *lsd1*, *acd5*, *acd6* and *acd11* [12].

Arabidopsis *cat2* mutants, deficient in *CATALASE2*, develop lesions that are day length and light intensity dependent [13–16]. PCD in *cat2* can be prevented by introduction of a loss of function mutation in the main SA biosynthesis enzyme ISOCHORISMATE SYNTHASE1 (ICS1) (mutations in *ICS1* are known as *sid2 - salicylic acid induction deficient2)* or by external supply of myoinositol, which repress *ICS1* transcript accumulation [17, 18]. PCD in *cat2* is long day-length dependent and is associated with changes in the glutathione redox status [13] but not in the ascorbate pool [16]. However, these responses are prevented when *cat2* plants are grown at high CO_2_, which suppresses photorespiration and hence H_2_O_2_ production. In addition to lesion formation, the *cat2* mutant also has an altered gene expression profile that includes genes related to SA and JA signaling [13, 18, 19]. Interestingly, altered expression of SA and JA marker genes is dependent on glutathione, the *cat2 cad2* (*cadmium sensitive2*) double mutant with reduced glutathione biosynthesis, showed repression in SA and JA-associated gene expression [19, 20]. The target of glutathione in relation to SA signaling could be NPR1 (NONEXPRESSER OF PR GENES 1), but other as yet unidentified components may also be involved [19].

Lesion formation in *cat2* is easily initiated through changes in growth conditions. In contrast to other LMMs, the exact source of initiating cell death in *cat2* is also clear – increased H_2_O_2_ production in the peroxisome. Hence, *cat2* can be used as a tool to understand the role of H_2_O_2_ in regulation of PCD. Here, we use *cat2* to study the role of defense and hormone signaling in regulation of PCD employing a collection of 56 double and triple *cat2* mutants. Through comparison of *cat2* with other LMMs we are able to identify additional biological mechanisms involved in PCD regulation and establish a core set of PCD regulators.

## Results and discussion

The *cat2* mutant is a convenient tool to explore the role of H_2_O_2_ in regulation of PCD [15]. To systematically delineate the signal pathways downstream from ROS we used a genetic approach and crossed mutants related to plant hormones (ABA, JA, SA, ethylene and auxin) and defense signaling pathways into *cat2* background (see Additional file 1: Table S1 for an overview of the genotypes used). Some of the *cat2* double mutants used in this work have been independently generated and characterized in other reports [16, 18–20]. Most of these double mutants display the same phenotypes as in this study with some subtle differences. While *cat2 sid2* [18] and *cat2 apx1 (ascorbate peroxidase1)* [21] had fully suppressed lesion formation in previous publications, in our growth conditions these double mutants had only partially suppressed lesion formation. Given that the *cat2* phenotype is dependent on growth conditions, including photoperiod, light intensity, and likely also other factors such as humidity or soil nutrient content, some differences could be expected.

### *In vitro* assay versus soil-grown plants

**Fig. 1:**
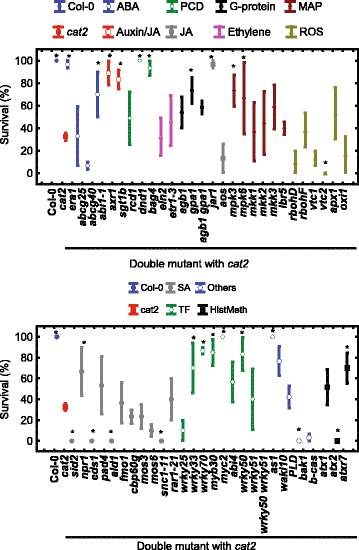
Survival of *cat2* and *cat2* double mutants in the *in vitro* assay. Fourteen-day old seedlings were transferred from SD (8 h/16 h) to LD (12 h/12 h) and gas exchange was restricted by sealing of plates with parafilm. Survival was scored after 8 days. *Asterisks* indicate significant difference from *cat2* single mutant at the end of LD treatment (*n* = 30; *p* <0.05, Fisher LSD). For a version of this figure with a time course of survival, see Additional file 2: Figure S1

Two main assays were used to screen the mutant collection: (1) survival of *in vitro* grown plants on plates after restriction of gas exchange and altered day length, (2) lesion formation in 4 week old soil-grown plants. For restriction of gas exchange, the *in vitro* grown plants (14 days old) were transferred from short day (SD, 8/16 h day night) to photorespiration-inducing condition (long day; LD, 12/12 h) by sealing plates with parafilm to limit air influx. A similar assay but with different light conditions has been extensively used to study photorespiration and *cat2* [22, 23]. The MS-agar media did not contain sugar, since in our growth conditions MS-media with sugar led to suppression of the *cat2* phenotype, presumably due to reduced photosynthesis and repression of photorespiration (Additional file 2: Figure S1). The extent of cell death was evaluated during 8 days after changes of photoperiod (Table 1; Fig. 1, Additional file 3: Figure S2). Double mutants that led to increased survival included *cat2 era1 (enhanced response to ABA1)*, *cat2 axr1 (auxin resistant1)*, *cat2 sgt1b*, *cat2 dnd1 (defense no death1)* and *cat2 as1 (asymmetric leaves1)*. Decreased survival was found in double mutants with defects in SA accumulation or SA signaling, *cat2 sid2*, *cat2 eds1 (enhanced disease susceptibility1)*, *cat2 ald1 (AGD2-like defense response protein1)* and ascorbic acid biosynthesis *cat2 vtc1 (vitamin c defective1)* and *cat2 vtc2* (Fig. 1).

**Table 1:** Quantification of *cat2* and *cat2* double mutant survival and lesion formation

Functional group	Genotypes	Survival (%)	*p*	Lesion (%)	±SE	*p*	Reference to single mutant
	Col-0	100.0	*	0	0	*	
	*cat2*	30.0		5.8	0.3		[16]
	Double mutants with *cat2*						
Abscisic acid	*era1*	96.7	*	0	0	*	[56]
	*abcg25*	32.9		2.4	1.2	*	[99]
	*abcg40*	6.7		3.0	1.6	*	[100]
	*abi1-1*	70.0	*	2.7	2.2	*	[101]
Auxin/Jasmonic acid	*axr1*	88.9	*	0	0	*	[51]
	*sgt1b*	83.3	*	0.9	0.3	*	[53]
Cell death regulation	*rcd1*	48.8		0	0	*	[34]
	*dnd1*	100.0	*	0	0	*	[30]
	*bag4*	93.3	*	7.4	3.2		[102]
Ethylene	*ein2*	32.5		6.8	2.6		[103]
	*etr1-3*	46.7		6.4	1.7		[104]
G-protein	*agb1*	53.9		2.7	0.5	*	[105]
	*gpa1*	73.3	*	2.4	0.7	*	[105]
	*agb1 gpa1*	58.5		0.9	0.5	*	
Jasmonic acid	*jar1*	96.7	*	7.9	1.4	*	[106]
	*aos*	13.3		5.1	2.2		[107]
MAP kinase signaling	*mpk3*	73.3	*	3.9	1.5		[108]
	*mpk6*	66.7	*	3.5	1.4	*	[108]
	*mkk1*	36.7		7.3	1.4		[108]
	*mkk2*	44.4		5.5	2.7		[108]
	*mkk3*	58.9		2.5	0.7	*	[108]
	*ibr5*	40.0		3.6	1.1	*	[109]
ROS biosynthesis/scavenger	*rbohD*	10.0		3.1	1.6	*	[25]
	*rbohF*	38.3	*	8.0	1.3	*	[25]
	*vtc1-1*	13.3	*	0	0	*	[110]
	*vtc2-1*	0	*	3.1	2.4		[110]
	*apx1*	53.3		2.2	1.1	*	[111]
	*oxi1*	16.7		4.5	0.9		[112]
Salicylic acid/Defense	*sid2*	0	*	0.4	0.3	*	[113]
	*npr1*	66.7	*	1.4	0.6	*	[114]
	*eds1*	0	*	2.0	1.9	*	[115]
	*pad4*	53.3		3.1	1.2	*	[116]
	*ald1*	0	*	4.5	2.1		[38]
	*fmo1*	36.7		3.6	1.7		[37]
	*cbp60g*	23.3		5.4	4.4		[117]
	*mos3*	23.3		3.2	1.7	*	[40]
	*mos6*	10.0		3.3	1.9	*	[40]
	*snc1-11*	0	*	4.2	1.3		[41]
	*rar1-21*	40.0		1.5	1.1	*	[118]
Transcription factors	*wrky25*	10.0	*	8.6	1.9	*	[65]
	*wrky33*	70.0	*	3.0	1.5	*	[119]
	*wrky70*	86.7	*	2.1	1.3	*	[62]
	*myb30*	85.0	*	2.1	0.9	*	[67]
	*myc2*	100.0	*	2.9	1.2	*	[120]
	*abi4-1*	56.7	*	3.1	1.2	*	[121]
	*wrky50*	83.3	*	2.3	1.6	*	[122]
	*wrky51*	40.0		3.5	1.8		[122]
	*wrky50 wrky51*	28.9		4.2	1.2		
	*as1*	100.0	*	0	0	*	[64]
Histone methylases	*atx1*	51.7		2.5	0.9	*	[68]
	*atx2*	0	*	2.4	0.6	*	[68]
	*atxr7*	70.0	*	3.1	1.3	*	[123]
Others	*wakl10*	76.7	*	2.5	1.5	*	[124]
	*PLD*	42.2		3.9	1.8		[125]
	*bak1-4*	0	*	7.7	1.1	*	[35]
	*β-cas*	3.3		8.4	4.1		[126]

Survival was scored in a plate assay for a period of 8 days after transfer to LD and restricted gas exchange which promotes photorespiration. The survival score was estimated 8 days after transfer, for a time course of survival see Additional file 3: Figure S2. Asterisks indicate statistically significant difference of survival between *cat2* and double mutant during LD treatment (*p* <0.05, *n* = 30, General Linear model for testing interactions). Lesions area (%; mean ± SE) were measured from 4 week old LD soil-grown plants.

Significantly different results from *cat2* single mutant are denoted by asterisks (*p* <0.05; *n* = 30, Fisher LSD)

**Fig. 2:**
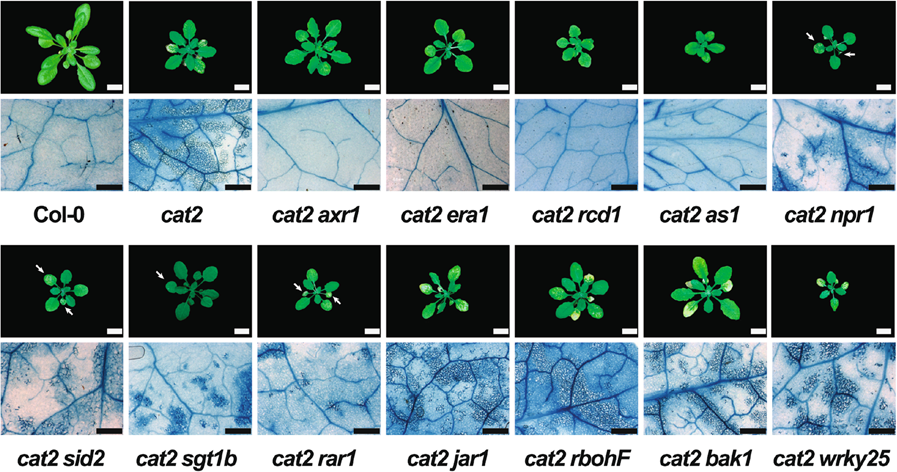
Formation of lesions in soil-grown *cat2* and *cat2* double mutants. Selected double mutants without lesions (*cat2 axr1, cat2 era1, cat2 rcd1, cat2 as1*), with few lesions (*cat2 npr1, cat2 sid2, cat2 sgt1b, cat2 rar1*) and with more lesions than *cat2* (*cat2 jar1, cat2 bak1, cat2 wrky25*). Cell death is indicated by trypan blue stain of 4 weeks old plants. *White scale bar* shows 1 cm and *black scale bar* 600 μm. All mutants used in this study are in Additional files 3, 4 and 5: Figures S2–S4

The restricted gas exchange assay used 2 week old *in vitro* grown seedlings. To assess the cell death response in soil-grown plants, lesion size was measured and cell death was visualized with trypan blue stain in 4 week old plants (Table 1, Fig. 2, Additional files 4, 5 and 6: Figures S3–S5). The two assays, restricted gas exchange versus lesion formation in soil grown plants, gave mostly similar results with some exceptions: *cat2 jar1 (jasmonate resistant1)* and *cat2 bag4* (*BCL-2-associated athanogene 4*) plants had high survival rate in restricted gas exchange, but displayed enhanced cell death in soil-grown plants compared to *cat2*. Contrary, *cat2 vtc1, cat2 atx2* (*trithorax protein like2*) and mutants with decreased SA accumulation *cat2 ald1*, *cat2 eds1* and *cat2 sid2*, died 8 days after transfer to restricted gas exchange although mature plants had no or few visual lesions, respectively (Table 1, Fig. 2, Additional files 4, 5, 6 and 7: Figures S3–S6).

In conclusion, both assays gave consistent results, with a few important exceptions. Ascorbic acid and SA double mutants were sensitive in restricted gas exchange but tolerant in soil-grown plants, conversely the *cat2 jar1* and *cat2 bag4* exhibited better performance *in vitro*. One additional factor that distinguishes the restricted gas exchange plate assay from the soil grown plants is temperature. Although the plates were incubated in a temperature controlled growth cabinet, the sealing of the plates led to an increased temperature and humidity inside the plates (2–3 °C and air humidity 98 %, measured with sensor for temperature and humidity DS1923, Digi-Key Corporation, Canada). In Arabidopsis high temperature alters the balance of defense signaling between SA and JA/ethylene, and in some LMMs the lesion formation is suppressed at higher growth temperature [24]. We speculate that some of the difference between restricted gas exchange and soil grown plants could be related to temperature and humidity, especially in SA mutants, and further study of *cat2* double mutants in different temperatures could give insight into cross talk between temperature and H_2_O_2_ signaling.

In the following sections we will highlight some of the double mutant data in relation to the functional category of the double mutants.

### The role of ROS production and ROS scavenging

A major source of extracellular ROS in plants is the NADPH oxidase, which are encoded by *RBOH (RESPIRATORY BURST OXIDASE HOMOLOGUE)* genes [25]. *RBOHD* and *RBOHF* are the main *RBOH* genes expressed in leaves and have been extensively characterized for their role in stress responses [26]. In avirulent pathogen infection *RBOHF* contributes more to cell death regulation than *RBOHD* [25]. The function of RBOH proteins may also be regulated by hetero-trimeric G-proteins [27]. In soil-grown plants *cat2 rbohF* but not *cat2 rbohD* had more lesions than *cat2* (Table 1, Figs. 1 and 2) consistent with a previous report [28]. Furthermore, reduced lesion formation was observed in alpha and beta subunits of the heterotrimeric G-protein *cat2 agb1, cat2 gpa1* and *cat2 agb1 gpa1* (Table 1). Hence, a ROS signal from RBOHF may be required to restrict the spread of cell death.

The *cat2* mutation impairs H_2_O_2_ scavenging, thus we expected that further removal of the important ROS scavenger ascorbic acid would lead to enhanced damage. This was indeed the case for *cat2 vtc1* and *cat2 vtc2* when assayed with restricted gas exchange (Table 1). In contrast, soil-grown *cat2 vtc1* had abolished visible lesion formation (Table 1, Additional file 7: Figure S6). The *vtc1* mutant is defective at an early step of ascorbic acid biosynthesis and in addition to lower ascorbic acid content also lacks biosynthesis of intermediates in cell-wall polysaccharide synthesis. Furthermore, *vtc1* has a pleiotropic phenotype including spontaneous cell death (Additional file 7: Figure S6; [29]). Thus, we suggest that in adult plants the *vtc1* mutation leads to activation of plant defenses that protect against damage from *cat2* induced H_2_O_2_ production.

### The role of cell death regulators

The *defense no death1* (*dnd1*) mutant is a conditional LMM, where *dnd1* exhibit spontaneous cell death in some growth conditions (Additional file 7: Figure S6) [30, 31]. In *cat2 dnd1* there was a reduced amount of lesions and increased survival compared to *cat2* (Table 1, Additional file 7: Figure S6). The exact step at which *dnd1* regulates cell death is currently unknown. However, the protein encoded by DND1, CYCLIC NUCLEOTIDE GATED CHANNEL2 (CNGC2), regulates Ca^2+^ transport and activation of nitric oxide signaling pathways [32]. Since Ca^2+^ is required for execution of cell death in catalase silenced tobacco [33], it is possible that DND1/CNGC2 mediates a Ca^2+^ signal required for initiation of cell death.

The *radical-induced cell death1* (*rcd1*) mutant is sensitive to apoplastic ROS, but tolerant to chloroplastic ROS induced damage caused by treatment with methyl viologen [34]. Likewise, intracellular ROS from *cat2* did not cause lesions in *cat2 rcd1* (Fig. 2). Thus, plants are able to distinguish the source and location of ROS production (apoplast versus chloroplast and peroxisome) and RCD1 acts as a central regulator to determine the switch from life to death [34].

BAK1 (BRI1-ASSOCIATED RECEPTOR KINASE) is a co-receptor of multiple receptor like kinases (RLK) and a regulator of cell death [35]. In *cat2 bak1-4* there was increased sensitivity to restricted gas exchange and increased lesion formation is soil-grown plants (Table 1, Figs. 1 and 2). BAK1 may regulate cell death responses through its interaction with BIR1 and regulation of ROS production [35, 36]. Thus, signaling via BAK1 could be required to restrict PCD in *cat2* through regulation of a ROS signal.

### The role of salicylic acid and TIR-NB-LRRs

Increased ROS production leads to increased biosynthesis of all major defense-related hormones – JA, SA, ethylene and ABA. SA is a crucial regulator of multiple plant defenses, and at high concentrations promotes cell death [12]. The amount of cell death in *cat2* is reduced when crossed the SA biosynthesis mutant *sid2* (Fig. 2). Several proteins are required for systemic accumulation of SA and function upstream and/or in parallel to SA and include ENHANCED DISEASE SUSCEPTIBILITY 1 (EDS1), PHYTOALEXIN DEFICIENT 4 (PAD4), AGD2-LIKE DEFENSE RESPONSE PROTEIN 1 (ALD1) and FLAVIN-DEPENDENT MONOOXYGENASE 1 (FMO1) [37, 38]. These regulators were also found as mutants that suppress cell death [39]. Lesion formation was reduced in *cat2 eds1* and *cat2 pad4* (Table 1), but given the multitude of signal pathways where EDS1 and PAD4 are involved, it is not straightforward to pinpoint exactly at which point these proteins regulate cell death.

Downstream from SA*,* NONEXPRESSOR OF PR GENES1 (NPR1) is required for execution of many SA responses. Loss of function in *npr1* attenuated cell death in *cat2* (Table 1; Fig. 2; [19]). Several other regulators of SA and defense signaling have been identified through mutant screens for altered defense responses and include the dominant mutant *suppressor of npr1-1, constitutive 1* (*snc1*) that has a mutation in a TIR-NB-LRR (Toll Interleukin1 receptor-nucleotide binding-Leu- rich repeat) and *mos3* and *mos6* (*modifiers of snc1*) that encode components of the nucleocytoplasmic trafficking machinery [40]. A loss of function allele in *snc1-11* restore normal growth to several LMMs; *mkp1* (*map kinase phosphatase1*), *srfr1* (*suppressor of RPS4-RLD1*) and *nudt6 nudt7* (*nudix hydrolase homolog6 nudix hydrolase homolog7*) directly showing that an appropriate level of signaling through NB-LRR proteins regulates cell death [41–43]. Indeed, several defense responses is regulated by the stability of NB-LRRs through RAR1 (REQUIRED FOR MLA12 RESISTANCE 1) [44]. Of the different mutations that have an impact on defense signaling down-stream or in parallel to SA, the *cat2 mos3* and *cat2 mos6* had fewer lesions than *cat2* indicating that execution of cell death requires nuclear localization of a yet to be identified cell death regulator (Table 1). Furthermore, *cat2 rar1* also had fewer lesions supporting a role for NB-LRR signaling in cell death execution [40–44]. Overall, using both a SA biosynthesis mutant and multiple mutants for regulators of SA accumulation, SA signaling and defense signaling, give support for the crucial role of SA as a regulator of cell death.

### The role of ethylene, jasmonic acid and auxin

Like SA, the role for JA and ethylene in regulation of cell death is complex. In different LMMs JA and ethylene can act as either positive or negative PCD regulators [12, 31]. Impaired ethylene signaling did not modulate defense reactions in *cat2*, since *cat2 ein2* and *cat2 etr1-3* survival rate and lesion development did not differ from *cat2*.

In response to biotic and abiotic stress several oxylipins are produced, where jasmonic acid (JA) is the best characterized. However, some of the pre-cursors of JA have biological activities of their own and for example 12-oxo-phytodienoic acid (OPDA) is perceived through its own receptor CYCLOPHILIN 20–3 to regulate cysteine biosynthesis and the cellular redox status [45]. The *aos* (*allene oxide synthase*) mutant is deficient in both OPDA and JA biosynthesis and in *jar1* (*jasmonate resistant1*) the formation of the biologically active JA-Ile is deficient [46]. However, *jar1* is not completely blocked in JA signaling; while *aos* is male sterile, *jar1* is fully fertile suggesting redundant pathways in the final step of JA-Ile biosynthesis [46]. Cell death in *cat2 aos* was similar to *cat2*, indicating a limited role for JA in ROS induced cell death (Table 1). However, *cat2 jar1* had increased amount of cell death in soil grown plants (Table 1, Fig. 2). Since the main difference between these mutants is that *aos* also lacks OPDA, the contrasting results for *cat2 aos* and *cat2 jar1* could indicate distinct roles for OPDA and JA in H_2_O_2_ induced cell death. A differential role between JA and OPDA has also been observed in the *flu* mutant where cell death is initiated through singlet oxygen production [47]. In *flu*, cell death is reduced when JA biosynthesis is abolished, but when both OPDA and JA biosynthesis were removed through *aos* no impact on cell death was observed [47]. Thus, both in H_2_O_2_ and singlet oxygen induced cell death, OPDA and JA appear to have distinct roles. Alternatively, the specific timing of biosynthesis of biologically active JA-Ile could be important for execution of PCD. Further investigation of other JA mutants, e.g. the JA receptor *coi1* could help clarify the role of JA in PCD regulation.

Auxin is a central regulator of plant development. Recently auxin has also been identified as an important regulator of stress responses, including PCD regulation in *cat2* [23] and mitochondria retrograde signaling [48, 49]. The auxin insensitive mutant *axr1* (*auxin resistant1*) is also JA insensitive [50]. AXR1 encodes a protein involved in regulation of the activity of Skp1-Cullin-F-box (SCF) complexes, which in turn regulate protein degradation [51]. Both the JA receptor COI1 and the auxin receptor TIR1 are regulated through this mechanism, explaining the auxin and JA insensitivity of *axr1*. SGT1b is also a protein involved in regulation of SCF complexes, and *sgt1b* has multiple phenotypes including auxin insensitivity, altered pathogen responses and defense signaling, and regulation of cell death [52, 53]. SGT1b is also part of a complex with RAR1 and HSP90 (HEAT SHOCK PROTEIN 90) to ensure proper function of TIR-NB-LRRs [54]. The phenotypes of *cat2 axr1*, *cat2 rar1* and *cat2 sgt1b* were similar, high survival in restricted gas exchange assay and few lesions in soil-grown plants (Table 1, Figs. 1 and 2). Given the importance of protein degradation in all aspects of plant signaling and the multiple phenotypes of *axr1*, *sgt1b* and *rar1* it is difficult to pinpoint exactly where AXR1, RAR1 and SGT1b might regulate ROS induced PCD, however, auxin or JA might be involved.

### The role of abscisic acid

ABA is an important hormone in abiotic stress responses (cold, salt and drought), in biotic stress responses through interaction with SA, and as a regulator in high light signaling [55]. ABA signaling is blocked in *abi1-1* (*ABA insensitive1*). In *cat2 abi1* there was increased survival as well as fewer lesions in soil-grown plants (Table 1 and Fig. 1). A mutant in the beta subunit of farnesyl transferase, *era1* (*enhanced response to ABA1*), has multiple phenotypes including increased sensitivity to ABA, altered developmental, stress and defense responses [56, 57]. Protein farnesylation is modification to proteins to change their subcellular localization . The *cat2 era1* double mutant was resistant to restricted gas exchange and did not develop lesions in soil-grown plants (Table 1, Figs. 1 and 2), thus a regulator of PCD might be farnesylated for proper function.

### The role of transcription factors and chromatin environment

ROS induced cell death caused by ozone treatment requires changes in gene expression since the extent of cell death can be decreased with a transcriptional inhibitor [58]. When bZIP10, a positive regulator of cell death [59], was knocked out in *cat2 bzip10,* a reduced amount of cell death was observed [18]. The *cat2* lesion phenotype was also suppressed by *cpsf30* (*cleavage and polyadenylation specificity factor30*), which regulates mRNA polyadenylation [60], thus both transcription and proper mRNA processing are regulatory steps in the execution of cell death. Analysis of gene expression signatures in response to several ROS treatments suggests the involvement of several TFs in regulation of ROS responses [61], hence TFs other than *bZIP10* are likely involved in the regulation of cell death in *cat2*.

Many TF mutations reduced cell death in *cat2* (*abi4-1*, *as1*, *myb30*, *myc2*, *wrky33* and *wrky70*) or led to enhanced cell death (*wrky25*) (Table 1, Figs. 1 and 2). Since several of these TFs either regulate JA responses (AS1, MYC2, WRKY70; [62, 63], SA responses (WRKY70; [62]), pathogen responses (AS1, WRKY25, WRKY33, WRKY70; [64–66]) or cell death (MYB30; [67]), this reinforces the importance of SA and JA in regulation of gene expression required for execution of PCD in *cat2*. To regulate gene expression TFs needs access to DNA, which is affected by how tightly DNA is packed in histones and chromatin. Histones can be post-translationally modified, including methylation by ARABIDOPSIS TRITHORAX1 (ATX1) [68]. Lesion development was decreased in *cat2 atx1*, *cat2 atx2* and *cat2 atxr7* indicating a regulatory role for histone methylation and chromatin packaging in cell death regulation (Table 1).

At a first glance it might appear counter-intuitive that such a large number of TFs would influence cell death regulation. However, high resolution TF-promoter interaction assay for genes encoding components related to the cell wall identified on average five TFs bound to each promoter [69]. Assuming that genes encoding proteins involved in PCD would have a similar promoter-TF relationship, we could expect that there are even more TFs than the ones identified here to be part of PCD regulation.

### The role of MAP kinase signaling

Mitogen-activated protein kinase (MPK) cascades regulate many aspects of development and stress responses in plants [70]. MAP kinase kinase kinases (MPKKKs) activate MAPK kinase kinases (MPKKs) that in turn activate MAP kinase (MPKs) that phosphorylate and activate target proteins, including TFs like WRKY2, WRKY25, WRKY33, WRKY34 and ERF6 [71, 72]. The *cat2 mpk3* and *cat2 mpk6* double mutants had increased survival in restricted gas exchange and *cat2 mpk6* exhibited fewer lesions in soil-grown plants (Table 1, Fig. 1, Additional file 4: Figure S3). MKK3 is one of MPKKs activating MPK6 [73], and *cat2 mkk3* also had a reduced amount of lesions. MKK3-MPK6 can phosphorylate MYC2 and regulate plant responses to blue light [74]; the similar phenotypes of *cat2 mkk3*, *cat2 mpk6* and *cat2 myc2* indicates that a similar signal pathway may take place during *cat2* lesion formation.

### Maximum photosynthetic efficiency

**Fig. 3:**
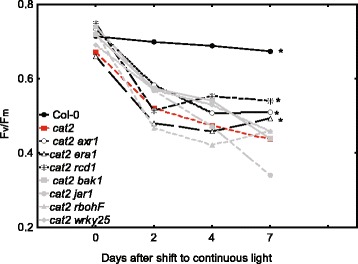
Maximum photosynthetic efficiency (F_v_/F_m_) in dark adapted plants after transfer to continuous light and restricted gas-exchange. Fourteen-days old seedlings were transferred from 70 μmol m^−2^ s^−1^ light intensity and 8 h day/16 h night to 120 μmol m^−2^ s^−1^ continuous light. F_v_/F_m_ was measured on shift day, 2^nd^, 4^th^, and 7^th^. The experiment was repeated three times using 20 plants per repeat (*n* = 60). *Asterisks* indicate significantly different F_v_/F_m_ values of double mutant in time scale and compared to *cat2* (*p* <0.05, General Linear Model)

Regulation of lesion size takes place at separate steps – lesion initiation, spreading and containment [75]. Since the restricted gas exchange and lesion development assays used rather long time scales, a separate method that follow early signaling in *cat2* could give information whether the increased tolerance of selected *cat2* double mutants were related to the initiation or the spreading phase. A sensitive assay to detect early stress symptoms is to measure the maximum photosynthetic efficiency of PSII (F_v_/F_m_) in dark adapted plants [23, 76]. In Arabidopsis photorespiration mutants, transferred from elevated CO_2_ to ambient CO_2_ conditions or from low to high light, a rapid onset of photoinhibition was accompanied by a decline in PSII maximum efficiency [77]. A subset of *cat2* double mutants with either strongly enhanced or reduced cell death were grown on plates for 14 days and assayed for photosynthetic efficiency over a period of 7 days after shifting to continuous light conditions (Fig. 3. NB, these growth conditions were different from the ones in Fig. 1 and Table 1). The PSII maximum efficiency declined dramatically in *cat2* compared to wild type, at the end of the experiment average values were 0.45 (*cat2*) and 0.75 (Col-0). For mutants with enhanced cell death (*cat2 bak1, cat2 jar1, cat2 rbohF, cat2 wrky25*); their F_v_/F_m_ decreased to 0.3, however, this did not differ significantly from *cat2*. Double mutants without lesions (*cat2 axr1, cat2 era1* and *cat2 rcd1*; Table 1, Fig. 2) were able to stabilize their photosynthetic efficiency and their F_v_/F_m_ were significantly higher than *cat2* 1 week after the shift to photorespiratory conditions (Fig. 3; for a version of Fig. 3 with error bars see Additional file 8: Figure S7). However, of these double mutants only *cat2 axr1* had stable photosynthetic efficiency at early time points during photorespiratory conditions and could indicate a role for AXR1 already in the early process of PCD regulation.

### H_2_O_2_ accumulation

H_2_O_2_ is a major byproduct of photorespiration and H_2_O_2_ is a regulator of PCD. Since *cat2* is deficient in H_2_O_2_ scavenging, we measured H_2_O_2_ concentration in 4 weeks old plants at 12:00 hours to further explore the mechanism underlying absent or increased lesion development in a subset of *cat2* double mutants. Our initial hypothesis was that double mutants with fewer lesions would have less H_2_O_2_ and vice versa for double mutants with increased cell death.

**Fig. 4:**
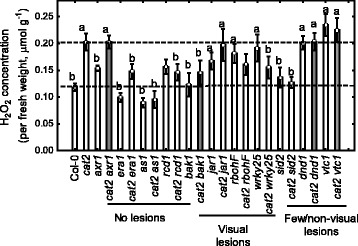
H_2_O_2_ concentration in *cat2* and selected double mutants in 4 weeks old plants measured with Amplex Red. Letter “a” indicates significant difference from Col-0 and letter “b” significant difference from *cat2* (mean ± SE, *n* = 6)

Most of the double mutants with decreased lesion formation also had less H_2_O_2_ (*cat2 era1, cat2 as1, cat2 rcd1* and *cat2 sid2*; Fig. 4). However, there were two exceptions, *cat2 axr1* and *cat2 dnd1*, that have no lesions but similar H_2_O_2_ content as the single *cat2* mutant. For double mutants with increased lesion formation (*cat2 jar1, cat2 rbohF* and *cat2 wrky25*) the H_2_O_2_ concentrations were the same as in *cat2.* Interestingly, *cat2 bak1* had reduced H_2_O_2_ production, even though *cat2 bak1* had more lesions than *cat2*. Overall this suggests that there is no strict correlation between H_2_O_2_ accumulation and cell death, instead as long as the initial steps towards PCD have been executed in *cat2*, further H_2_O_2_ accumulation appears to not influence the PCD process.

### Similarities between *cat2* and other mutants that develop spontaneous lesions

**Fig. 5:**
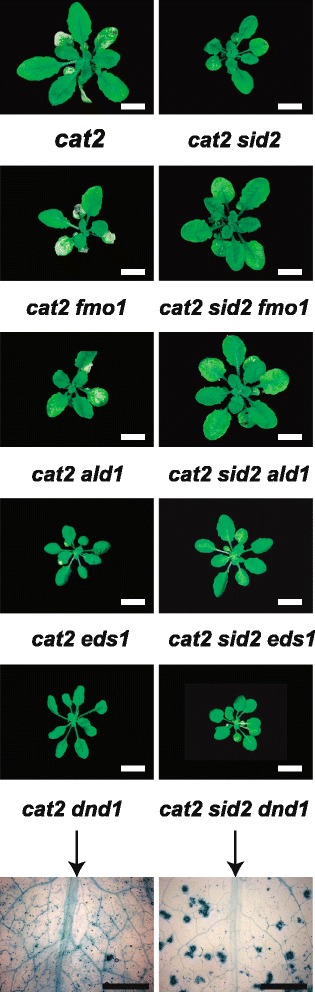
Lesion development in soil-grown *cat2* double and triple mutants. Cell death is indicated by trypan blue stain for weeks old plants. *White scale bar* shows 1 cm and *black scale bar* 600 μm

Arabidopsis mutants that spontaneously develop lesions, the LMMs, have long been used to study regulation of cell death and defense signaling [11, 12]. For three of these mutants extensive collections of double or triple mutants have been generated to identify regulators of cell death: *accelerated cell death6* (*acd6*) [78], *dnd1* [31] and *syp121 syp122* [39]. All three mutants *acd6*, *dnd1* and *syp121 syp122* have constitutively activated defense pathway and increased SA accumulation. We compared the regulators of PCD in *cat2* (Table 1) with the corresponding information from *acd6*, *dnd1* and *syp121 syp122* to determine whether cell death in *cat2* is unique or if there is a common genetic program for execution of cell death. In all four mutants SA plays a major role, mutations in *sid2*, *npr1*, *pad4* and *eds1* reduce the extent of cell death (Table 1; [31, 78, 39]. In *cat2*, *dnd1* and *syp121 syp122* the *rar1* mutation also led to less lesion formation (this mutant was not tested in *acd6*). In contrast *ald1* and *fmo1* mutations protected *acd6*, *dnd1* and *syp121 syp122*, but not *cat2*. Overall this emphasizes the role of SA in cell death execution and that *PAD4, EDS1, RAR1* and *SGT1B* are regulators of cell death downstream from activation of cell death by different mutations in *acd6*, *dnd1*, *syp121 syp122* and *cat2*. In our growth conditions the *sid2* and *eds1* mutations only led to partial reduction of the lesion formation in *cat2*, similarly *acd6*, *dnd1* and *syp121 syp122* were only partially rescued by these mutations. In contrast introduction of both *sid2* and *eds1* mutations led to increased plant health in *acd6*, *dnd1* and *syp121 syp122* compared to introduction of only one mutation. This prompted us to generate the *cat2 sid2 eds1, cat2 sid2 fmo1* and *cat2 sid2 ald1* triple mutants (Fig. 5). Although, *cat2 sid2 fmo1* and *cat2 sid2 ald1* had less lesions compared to *cat2 fmo1* and *cat2 ald1* the cell death area did not differ from *cat2 sid2*. Of the different triple mutants *cat2 sid2 eds1* completely lacked lesions, indicating the importance of SA and EDS1 acting together as regulators in execution of cell death. Indeed, inactivation of both SA (via the *sid2* mutation) and *eds1* leads to a compromised HR [79]. The *cat2 dnd1* double mutant represented a case where both of the single mutants are LMMs (Additional file 7: Figure S6). The amount of cell death was reduced in *cat2 dnd1* compared to *cat2* (Table 1). Since *sid2* suppress the extent of cell death in both *cat2* and *dnd1*, the triple mutant *cat2 sid2 dnd1* was tested (Fig. 5). Visible lesions were absent, but there were small patches of dead cells visualized with trypan blue stain in a pattern different compared to *cat2 dnd1* (Fig. 5). In future studies there will be a need to move to assays that more emphasize the behavior of specific cells rather than the average of whole leaves. We conclude that cell death in *cat2* is largely executed through a similar genetic program as in other lesion mimic mutants, including the dependence on SA and EDS1.

### Gene expression analysis of *cat2* in comparison with cell death mutants

If cell death is executed through a common genetic program we would also expect to see a similar gene expression profile of *cat2* in comparison with other LMMs. We obtained array data from three studies with *cat2* performed on the Affymetrix ATH1 chip. Vanderauwera et al. [80] used plants silenced for catalase in a high light experiment with two time points, 3 and 8 h. Queval et al. [81] grew *cat2* in high CO_2_ followed by transfer to ambient CO_2_ in either short day or long days for 2 and 4 days. Sewelam et al. [82] grew *cat2* and transgenic GO5 lines in high CO_2_ followed by transfer to ambient CO_2_ (GO5 plants express GLYCOLATE OXIDASE in the chloroplast, these plants produce H_2_O_2_ in the chloroplast during photorespiratory conditions). These experimental designs allow the identification of H_2_O_2_ responsive transcripts after the shift to photorespiratory conditions. The array raw data was normalized and clustered with Bayesian hierchical clustering together with array data from LMMs and various other treatments that induce ROS production or cell death (see Methods for full list of experiments). The gene list used in the cluster analysis was the gene ontology category cell death (488 genes, Additional file 9: Table S2).

**Fig. 6:**
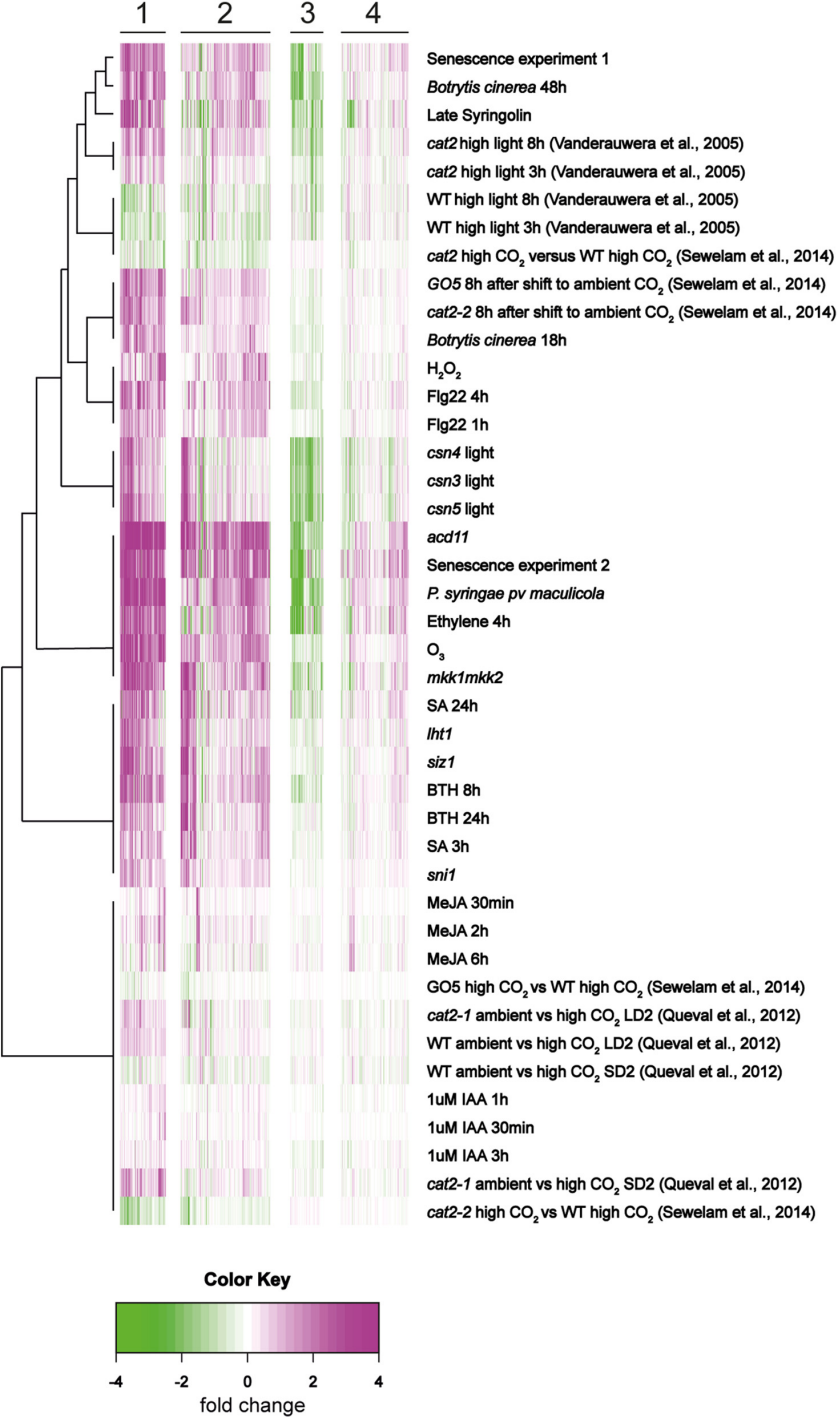
Cluster analysis of cell death related genes in *cat2* and other LMMs. Experiments performed on the Affymetrix ATH1 chip were obtained from *cat2*, LMMs, constitutive defense mutants, hormone treatments, senescence and biotic and abiotic stresses (see Methods for a full list of experiments). The gene ontology category cell death (488 genes) was used for Bayesian hierarchal clustering. Values are mean of log2 ratio of the treatment and control expressions. *Magenta* and *green* indicate increased and decreased expression compared with untreated or wild type plants, respectively

Four major clusters were obtained (Fig. 6). The Queval [81] experiment gave weaker changes in gene expression compared to the other experiments, possibly due to the use of a later time point (2 days versus 3 to 8 h for the other experiments). Cluster 1 contained genes with strongly induced gene expression in LMMs; these genes also had high expression in *cat2*, GO5, by ROS treatment (ozone), and during pathogen infection and senescence. The genes in this cluster (Additional file 9: Table S2) included genes that in our mutant analysis were important in lesion formation, including *SID2*, *PAD4*, *WRKY25* and *WRKY33*. Cluster 2 was more heterogeneous in terms of expression pattern, but had genes with increased expression in both cell death mutants, *cat2* and GO5 plants from the Sewelem [82] experiment. Cluster 3 contained genes with decreased expression and cluster 4 genes with minor altered expression by most treatments. From the hormone perspective, SA and BTH (Benzothiadiazole, a SA analog) gave a similar expression profile as *cat2* and other cell death mutants, reinforcing the crucial role of SA in regulation of cell death.

We conclude that the shift to photorespiratory conditions in *cat2* and promotion of H_2_O_2_ production leads to a gene expression profile that is also seen in plants undergoing cell death activated by either mutation or through treatments. Thus, both double mutant analysis in *cat2* and gene expression profiles, suggest that plants have a core program that includes SA and EDS1 for execution of PCD and that H_2_O_2_ may be one of the key signals of this program.

### Localized gene expression in *cat2*

**Fig. 7:**
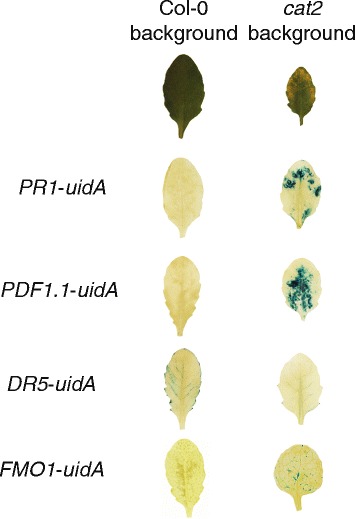
Local gene expression in *cat2*. Histochemical analyses of *uidA* expression in Col-0 and *cat2* in 4 week old soil-grown plants. Promoter-*uidA* lines included *PR1*, *PDF1.1*, *DR5* and *FMO1* (Table 2)

Microarray analysis performed with *cat2* was done with either whole rosettes or whole leaves [80–82]. This sampling strategy is likely to average out any cell specific responses across the whole leaf. In particular, it would be informative if genes related to PCD are expressed only at the lesions in *cat2*, or if altered expression is spreading outwards from the lesions. Here we crossed *cat2* with several promoter-*uidA* fusions, which allows GUS staining and visualization of gene expression activity at the site of lesion formation in *cat2* and surrounding tissues in 4 week old plants (Table 2, Fig. 7).

**Table 2:** Summary of promoter GUS line expression in Col-0 and *cat2* background

Reporter gene	Promoter is activated by	References
*PR1*-*uidA*	Salicylic acid	[127]
*DR5*-*uidA*	Auxin	[128]
*FMO1*-*uidA*	Local cell death	[129]
*PDF1.1*-*uidA*	ROS, pathogen infection	[86]

Expression of the auxin reporter *DR5-uidA* in *cat2* decreased in the whole leaf (Fig. 7), consistent with a previous report [83]. The lack of localized *DR5* expression at the site of lesions would suggest that auxin is not directly involved in the PCD process and could instead play a role in reprogramming of leaf growth in response to elevated ROS [83, 84]. A screen for chemicals that rescue the photoinhibition in *cat2* revealed that both auxin and synthetic auxin-like molecules can rescue *cat2* phenotypes [23]. Thus, mutations in auxin signaling components *axr1*, *sgt1b* and *as1* (Table 1) as well as application of auxin rescue *cat2* phenotypes, whereas *DR5* expression suggest decreased auxin signaling in *cat2*. Given the central role for auxin as a growth hormone it is possible that different branches of auxin signaling could be activated during development versus PCD or higher H_2_O_2_ levels. This has been observed in mitochondria retrograde signaling, where two specific AUXIN RESPONSE FACTORS (ARF7 and ARF19) act as negative regulators [49]. Introduction of other auxin reporter constructs in *cat2*, including DII-Venus [85] could help to unravel the role of auxin in the PCD process.

One of the most commonly used marker genes to study SA signaling is *PR1* (*PATHOGENESIS-RELATED GENE 1*). Expression of *PR1* was very high around lesions (Fig. 7), this could indicate that SA biosynthesis is initiated at the cells where PCD is taking place. Expression of *PDF1.1* (*PLANT DEFENSIN 1.1*) is high at sites of *B. cinerea* infection and by paraquat treatment [86]. In *cat2 PDF1.1* expression was high in leaves with lesions, but interestingly the signal was strongest some distance away from lesions, suggesting that expression of this gene is a systemic response. Expression of *FMO1* is induced in response to pathogens in an *EDS1/PAD4* dependent manner [37], by ROS treatment and during cell death [87]. Expression of *FMO1* was more localized in *cat2* than either *PR1* or *PDF1.1* (Fig. 7).

Overall the results using GUS lines clearly show that there is a spatial component to gene expression induced during lesion formation in *cat2*. Future studies in the context of gene expression should aim towards more cell specific assays, with the goal to find the genes directly related to lesion formation versus genes responsible for initiating defenses further away in the leaf and in systemic tissues.

## Conclusion

**Fig. 8:**
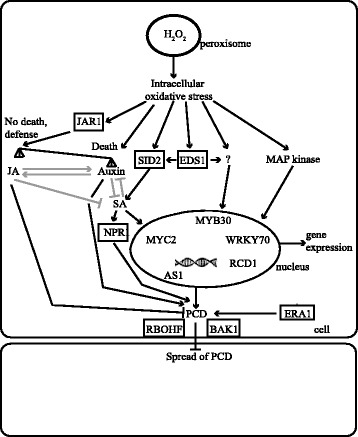
Model for ROS in cell death regulation. Intracellular H_2_O_2_ production caused by the *cat2* mutation lead to increased concentration of hormones (SA and JA, through SID2 and JAR1) and activation of multiple signaling pathways. The balance and timing of biosynthesis of auxin, SA and JA is one important determinant of cell death. In parallel, MPK signaling and other unknown signaling pathways may directly target TFs and regulate their activity and interaction with DNA with a subsequent change in gene expression. Other mechanisms in the nucleus also provide a suitable environment for accurate gene expression, including RCD1 that interacts with TFs. A yet to be identified regulator of cell death is farnesylated for proper function by ERA1. Extensive cross-talk between hormone signaling pathways allow fine tuning of the signal. Controlled ROS production via BAK1 and RBOHs may provide a signal to neighboring cells leading to propagation of cell death

Based on the data presented here and in previous research on *cat2* [15, 18, 21, 23, 28]; we suggest a model for signaling events leading to cell death in *cat2* (Fig. 8). After a certain threshold concentration of H_2_O_2_ has built up in *cat2* the lesion process is initiated. Subsequently two main pathways are activated: SA and its associated signaling partners including EDS1; and auxin/JA. These two pathways are likely to exhibit extensive crosstalk. Transcriptional regulation is essential for cell death control and includes several TFs (including AS1, WRKY33, WRKY70, MYB30 and MYC2) and proteins that interact with TFs (RCD1). The exact molecular function for other cell death regulators remain to be identified, as an example BAK1, a co-receptor of many plasma membrane receptors, is part of a larger complex that regulate ROS production via RBOHs [88]. The enhanced cell death of *cat2 bak1* and *cat2 rbohF* (Table 1), could hint towards a role for regulated apoplastic ROS production as a signal to inhibit the spread of cell death. Using assays that directly target protein-DNA interaction (chromatin immunoprecipitation) could help unravel which targets genes of AS1, MYB30 and WRKY70 may be involved in execution of cell death.

## Methods

### Plant material and growth conditions

All the mutants were in Columbia-0 (Col-0) background and Col-0 was used as control plant for all experiments. Arabidopsis mutants were obtained from NASC [130] or were gifts, see Additional file 1: Table S1 for more details [89, 90]. Double and triple mutants were constructed using *cat2* (SALK_076998) as the pollen acceptor. Double and triple mutants were screened for visible *cat2* mutant phenotypes (dwarf, bleached leaves, visual cell death) and subsequently genotyped using PCR based CAPS, dCAPS [91] and T-DNA markers (Additional file 1: Table S1). After screening of double/triple homozygotes in the F2 generation, all mutants were confirmed in F3 or F4 generations.

Sterilized seeds were placed on Murashige and Skoog (MS) medium (1/2 MS salts, and 0.7 % agar), stratified for 3 days and transferred to growth chamber. One week old plants were transplanted into pots with 1:1 peat:vermiculate mixture with one Col-0, one *cat2*, one of the single mutants and the corresponding *cat2* double mutant (4 plants per pot). Plants were subsequently grown on soil for 3 weeks in a controlled chambers (MCA1600, Snijders Scientific, Netherlands), at 21 °C/19 °C under a 12 h day/12 h night regime, light intensity 120 μmol m^−2^ s^−1^, and 70 % relative humidity.

For the *in vitro* plate assays, plants were grown on plates in 8 h day/16 h night during 14 days, subsequently sealed with parafilm M and transferred to 12 h/12 h regime for 8 days (survival estimation). Chamber temperature was kept 23 °C throughout day (Sanyo chambers, Sanyo Electric Co, Japan).

### Estimation of lesions and cell death

Four week old plants were photographed and visual lesion size was calculated from photos using Adobe Photoshop Professional CS5.1. The mutant collection was screened 6 times with 5 plants per repeat for lesion size. Fully expanded leaves were used for trypan blue staining to confirm cell death [92].

Survival during restricted gas exchange was calculated from photos scoring dead plants per mutant. The mutant screen was repeated three times with 10 plants per repeat.

### Photosynthetic performance

Plants were grown on plates at 70 μmol m^−2^ s^−1^ for 2 weeks in SD (8 h/16 h), subsequently sealed with parafilm (no air influx) and transferred to continuous light (120 μmol m^−2^ s^−1^ irradiance) to promote photorespiration. Chlorophyll fluorescence was measured during 7 days (on shift day, 2^nd^, 4^th^ and 7^th^ day). Using Imaging PAM-2000 (Heinz Walz GmbH, Germany), the photosystem II (PSII) maximum efficiency (F_v_/F_m_) was determined in which F_v_ denotes variable fluorescence (ability of PSII to perform photochemistry) and F_m_ maximal fluorescence (PSII centers closed).

### Histochemical analyses and H_2_O_2_ quantification

GUS staining was performed according to [93]. For H_2_O_2_ quantification 100 mg of leaf material was collected from 4 weeks old plants and frozen in liquid nitrogen at 12:00 when CATALASE2 expression and photorespiration are presumably at their highest level [6, 8]. Frozen samples were milled using Silamat S6 (Ivoclar Vivadent, NY, USA) and extracted with 500 μl of 50 mMK_2_HPO_4_, pH 7.2. The slurry was cleared by centrifugation at 16,000 g for 15 min at 4 °C, and 50 μl of supernatant was incubated with 0.1 mM Amplex Red (Thermo Fisher Scientific Inc., MA, USA) and 0.2 mM Horseradish peroxidase at room temperature for 30 min [94]. Absorbance was detected at 570 nm wavelength (Multiskan FC, Thermo Scientific Inc., MA, USA). Calibration curves were calculated in 0, 5, 10, 25, 50 and 75 μM H_2_O_2_ range.

### Analysis of gene expression array data

Affymetrix raw data were processed with robust multiarray average normalization using Bioconductor limma and affy packages in R [95, 96]. Raw data from the Affymetrix ATH1-121501 platform was obtained from several data sources: NASC Arrays [131] (BTH, NASCARRAYS-392; Senescence experiment 1, NASCARRAYS-52; Senescence experiment 2, NASCARRAYS-150; SA, NASCARRAYS-192). ArrayExpress [132] (MeJA, E-ATMX-13; Syringolin, E-MEXP-739; catalase deficient Arabidopsis and high light, E-MEXP-449) Gene Expression Omnibus [133] (H_2_O_2_, GSE5530; *csn3*, *csn4* and *csn5*, GSE9728; *lht1*, GSE19109; *mkk1mkk2*, GSE10646; *sni1*, GSE6827; *siz1*, GSE6583; SA 24 h, GSE14961; Ethylene, GSE14247; Flg22, GSE5615; *Botrytis cinerea*, GSE5684; Pseudomonas syringae ES4326, GSE18978; ozone, GSE5722; auxin, GSE39384; *cat2* and GO5, GSE54534; *cat2* and daylength, GSE27985). Raw data for *acd11* were obtained from John Mundy. Gene expression for each experiment was computed by log2 fold changes between treatment and control, or between wild type and mutant. The processed data was discretized and clustered using Bayesian Hierarchical Clustering [97]. Bootstrap analysis was done as previously described in [98].

### Statistical analyses

One-way ANOVA was used to test differences in survival, PCD and H_2_O_2_ between mutants (Statistica 7.1, Stat Soft Inc., OK, USA). After confirmation of statistically significant F-value, the pair-wise comparison between *cat2* and double mutants was performed with Fisher LSD test. Analysis of variance (GLM procedure) was used for multiple comparisons between mean values of factor levels to find significant interactions, thus, the effect was tested for categorical predictor variables (time from shift, *cat2* versus double mutant in binary system) with single dependent variable (survival or F_v_/F_m_).

## Additional files

Additional file 1: Table S1. Primers and restriction enzymes used for mutant genotyping. Additional information about the mutants used. (XLSX 14 kb) Additional file 2: Figure S1. The *cat2* phenotype depends on sucrose concentration in the agar media. Ten days old seedlings in different sucrose concentrations (0, 0.5, 1 and 2 %). At 0 % sucrose the seedlings were bleached, with increased sucrose concentration the seedlings were green. (TIFF 2004 kb) Additional file 3: Figure S2. Survival of *cat2* and *cat2* double mutants in the *in vitro* plate assay. Fourteen-days old seedlings were transferred from SD (8 h/16 h) to LD (12 h/12 h) and survival was scored on the 1^st^, 5^th^, 8^th^ days after shift to LD. The experiment was repeated three times using 10 plants (mean±SE, *n*=30). (EPS 14277 kb) Additional file 4: Figure S3. Lesion formation in *cat2* and *cat2* double mutants in 4 week old soil-grown plants in LD (12 h/12 h). Cell death is indicated by trypan blue stain. White scale bar shows 1 cm and black scale bar 600 μm. The experiment was repeated six times using 5 plants per repeat. (EPS 36576 kb) Additional file 5: Figure S4. Lesion formation in *cat2* and *cat2* double mutants in 4 week old soil-grown plants in LD (12 h/12 h). Cell death is indicated by trypan blue stain. White scale bar shows 1 cm and black scale bar 600 μm. The experiment was repeated six times using 5 plants per repeat. (EPS 36989 kb) Additional file 6: Figure S5. Lesion formation in *cat2* and *cat2* double mutants in 4 week old soil-grown plants in LD (12 h/12 h). Cell death is indicated by trypan blue stain. White scale bar shows 1 cm and black scale bar 600 μm. The experiment was repeated six times using 5 plants per repeat. (EPS 30832 kb) Additional file 7: Figure S6. Microscopic cell death in *dnd1*, *vtc1* and corresponding double mutants with *cat2*. Cell death is indicated with trypan blue stain. White scale bar shows 1 cm and black scale bar 600 μm. (EPS 23350 kb) Additional file 8: Figure S7. Maximum photosynthetic efficiency (F_v_/F_m_; mean±SE) in dark adapted plants after transfer to continuous light and restricted gas-exchange. Fourteen-days old seedlings were transferred from 70 μmol m^−2^ s^−1^ light intensity and 8h day/ 16h night to 120 μmol m^−2^ s^−1^ continuous light. F_v_/F_m_ was measured on shift day, 2^nd^, 4^th^, and 7^th^. The experiment was repeated three times using 20 plants per repeat (*n*=60). Asterisks indicate significantly different F_v_/F_m_ values of double mutant in time scale and compared to *cat2* (*p*<0.05, General Linear Model). (EPS 815 kb) Additional file 9: Table S2. A list of the genes used in the *cat2* gene expression cluster analysis. (XLSX 15 kb)

## Abbreviations

ABA: Abscisic acid
F_v_/F_m_: Photosystem II maximum photosynthetic efficiency
HR: Hypersensitive response
JA: Jasmonic acid
H_2_O_2_: Hydrogen peroxide
LD: Long day
LMM: Lesion mimic mutant
MPK: Mitogen-activated protein kinases
MS: Murashige and Skoog medium
OPDA: 12-oxo-phytodienoic acid
PCD: Programmed cell death
RBOHs: Respiratory burst oxidase homologs
ROS: Reactive oxygen species
SA: Salicylic acid
SD: Short day
TF: Transcription factor
