# Supplementary figures and images for: A genetic framework for H2O2 induced cell death in Arabidopsis thaliana
Source: BMC Genomics. 2015 Oct 23;16:837. doi: 10.1186/s12864-015-1964-8 (PMC4619244; doi:10.1186/s12864-015-1964-8)

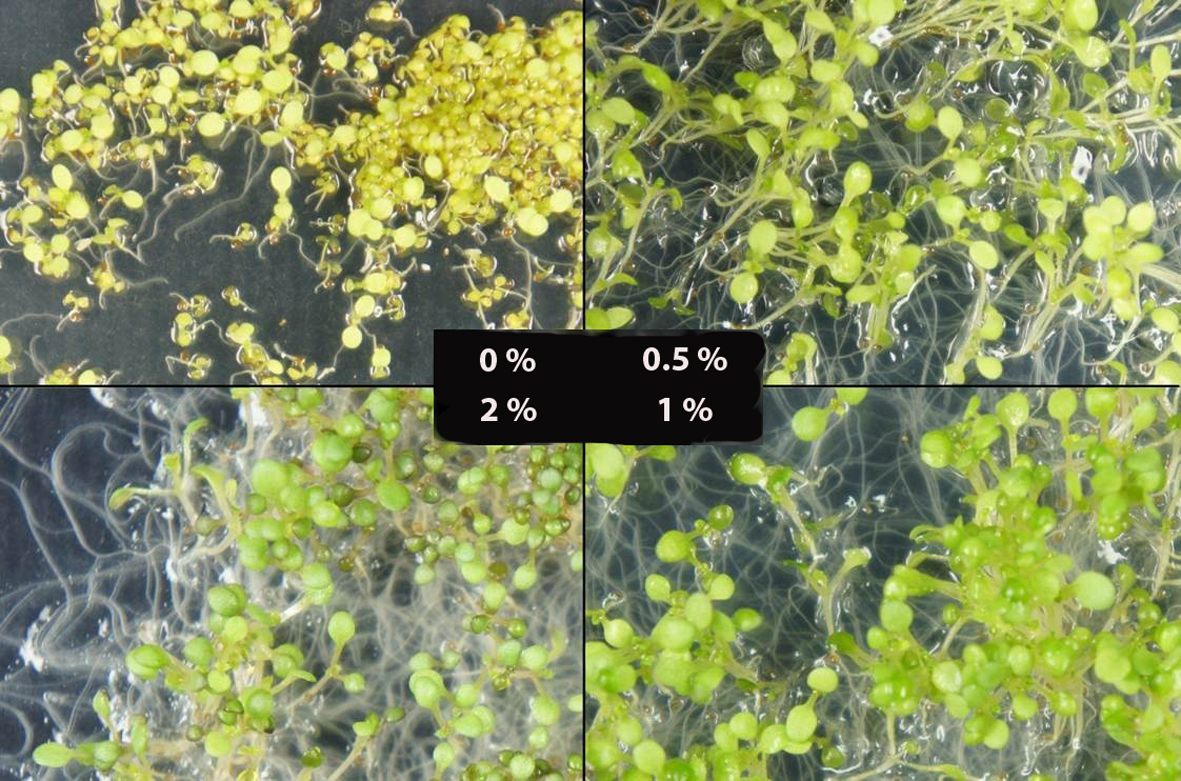

Supplement: Additional file 2: Figure S1. — The cat2 phenotype depends on sucrose concentration in the agar media. Ten days old seedlings in different sucrose concentrations (0, 0.5, 1 and 2 %). At 0 % sucrose the seedlings were bleached, with increased sucrose concentration the seedlings were green. (TIFF 2004 kb) [file 12864_2015_1964_MOESM2_ESM.tif]
